# O.4.3-3 Optimising time-use data to determine the optimal balance between 24-hour movement behaviours for the prevention of childhood obesity

**DOI:** 10.1093/eurpub/ckad133.189

**Published:** 2023-09-11

**Authors:** Aleš Gába, Charlotte Lund Rasmussen, Ty Stanford, Jan Dygrýn, Dorothea Dumuid, David Janda, Karel Hron

**Affiliations:** Palacký University Olomouc, Olomouc, Czech Republic; Curtin University, Perth, Australia; University of South Australia, Adelaide, Australia; ales.gaba@upol.cz; Palacký University Olomouc, Olomouc, Czech Republic; University of South Australia, Adelaide, Australia; ales.gaba@upol.cz; Palacký University Olomouc, Olomouc, Czech Republic; Palacký University Olomouc, Olomouc, Czech Republic

## Abstract

**Purpose:**

This cross-sectional study aimed to investigate the associations between 24-hour movement behaviours and adiposity and to determine optimal durations for sleep, sedentary behaviour (SB), light physical activity (LPA), and moderate-to-vigorous physical activity (MPVA) for preventing childhood obesity.

**Methods:**

The study involved 659 Czech children and adolescents aged 8 to 18 years, whose 24-hour movement behaviours were measured using wrist-worn accelerometers. Data on sleep duration and time spent in SB, LPA, and MPVA were derived from raw acceleration data recorded for seven consecutive days. Healthy adiposity status was defined as any value of the adiposity indicator below the 85th percentile. The study employed compositional regression analysis to investigate the association between movement behaviour composition and adiposity. An 'optimal day' was defined as the compositional mean of all compositions that were associated with a healthy adiposity status.

**Results:**

We found a significant association between the composition of movement behaviours and visceral adipose tissue (F = 3.672, *p* = 0.013) in children and the percentage of fat mass (F = 2.733, *p* = 0.044) in adolescents. The optimal durations of movement behaviours differed across various age groups. For children, the optimal balance of movement behaviours consisted of 8.5 hours of sleep, 10.8 hours of SB, 3.9 hours of LPA, and 0.8 hours of MVPA. For adolescents, the 'optimal day' consisted of 7.5 hours of sleep, 12.4 hours of SB, 3.5 hours of LPA and 0.5 hours of MVPA.

**Conclusions:**

It seems crucial to optimise the duration of sleep, SB, and physical activity of varying intensities to prevent excess adiposity. Additionally, age-specific variations should be considered while suggesting the most appropriate durations for 24-hour movement behaviours to prevent childhood obesity.

**Funding source:**

This research was funded by research grants from the Czech Science Foundation (18-09188S and 22-02392S).

